# Testing for mean and correlation changes in microarray experiments: an application for pathway analysis

**DOI:** 10.1186/1471-2105-11-60

**Published:** 2010-01-28

**Authors:** Mayer Alvo, Zhongzhu Liu, Andrew Williams, Carole Yauk

**Affiliations:** 1 Department of Mathematics and Statistics, University of Ottawa, Ottawa, ON K1N 6N5 Canada; 2 Environmental Health Science and Research Bureau, Environmental and Radiation Health Sciences Directorate, Health Canada, Ottawa ON K1A 0K9 Canada

## Abstract

**Background:**

Microarray experiments examine the change in transcript levels of tens of thousands of genes simultaneously. To derive meaningful data, biologists investigate the response of genes within specific pathways. Pathways are comprised of genes that interact to carry out a particular biological function. Existing methods for analyzing pathways focus on detecting changes in the mean or over-representation of the number of differentially expressed genes relative to the total of genes within the pathway. The issue of how to incorporate the influence of correlation among the genes is not generally addressed.

**Results:**

In this paper, we propose a non-parametric rank test for analyzing pathways that takes into account the correlation among the genes and compared two existing methods, Global and Gene Set Enrichment Analysis (GSEA), using two publicly available data sets. A simulation study was conducted to demonstrate the advantage of the rank test method.

**Conclusions:**

The data indicate the advantages of the rank test. The method can distinguish significant changes in pathways due to either correlations or changes in the mean or both. From the simulation study the rank test out performed Global and GSEA. The greatest gain in performance was for the sample size case which makes the application of the rank test ideal for microarray experiments.

## Background

DNA microarrays are powerful tools used for the analysis of genome-wide gene expression. The dimensionality of commercially available platforms has dramatically increased over the years. The technology has evolved rapidly and now provides a relatively accurate method to determine what genes are differentially regulated as a result of a particular condition. Although the technology is intended to provide a means to understand the response of a system as a whole, the interpretation of DNA microarray data has generally been carried out by analysis of individual genes for differential expression. With the broad goal of understanding the biology of the system, the evaluation of single genes is impractical.

Reducing the dimensionality of microarray data through the analysis of pathways or gene sets related to biological functions, instead of analysing individual genes, will facilitate deriving biologically meaningful experimental results. However, classical multivariate approaches are generally not appropriate statistical tools for the analysis of pathways because the numbers of samples in microarray experiments are often very small, generally ranging from three to ten per experimental condition. As such, it is difficult to ascertain the nature of the underlying distribution. In 2002, an approach using Gene Ontology (GO) was proposed that assigns genes into groups and looks for over-representation of differentially expressed genes within these sets [1,2]. Since that time over 20 such tools have been developed [3-10].

The Fisher's Exact Test is one of the most popular methods underlying most software investigating over-representation of genes from a gene list for pathways, terms or ontologies. However, the assumption that the probes within pathways are independent is not satisfied since genes within pathways are highly associated. Moreover, an over-representation approach, such as the Fisher's Exact Test, focuses only on the number of significantly expressed probes, but ignores the magnitude of changes of the fluorescence intensity.

The Gene set enrichment analysis (GSEA) [5] method is becoming more commonly used for pathway analysis. This technique, introduced by Moothe et al. [4] involves the application of GSEA to pre-determined gene sets to identify differences in expression between normal and diseased patients. The methodology was later modified by Subrammanian et al [5]. GSEA consists of ranking the genes on the microarray, g_1_, g_2_, ..., g_M_, by their signal-to-noise ratio(SNR),

Where and are the estimated mean and standard deviations of normalized signal intensity for sample *i*, *i = *1, 2.

Two empirical cumulative distribution functions are then calculated for each gene set, *G *as follows,

where *N*_*G *_represents the number of genes in the gene set *G*.

The difference between the two empirical cumulative distribution functions is calculated for each gene in the gene set. The maximum difference across all the genes in the gene set is taken to be the enrichment score. A permutation-based p-value is then calculated for each gene set which is used to identify significant alterations in expression across experimental conditions. A high enrichment score is achieved when a gene set contains a large number of highly ranked genes.

GSEA incorporates the magnitude of the gene fluorescence intensity values into its model. However, as discussed by Damian and Gorfine [11], GSEA is hindered by several factors. The primary concern is that the power of the test is a function of the number of genes in the pathway. Thus the method may not work well with small gene sets.

An alternative approach to examine pathway-associated effects is the Global test. This method was originally proposed to test correlation structures due to familial aggregation in pedigrees by Houwelingen et al. in 1995 [12,13]. This methodology applies a goodness-of-fit test for a generalized linear model having a canonical link function with a known dispersion parameter. In 2003, familial aggregation testing was adapted by Goeman et al. for microarray data analysis [8] and was designed to determine whether the common expression pattern of genes within a pre-defined set is significantly related to experimental condition. A generalized linear model is used to estimate a statistic for each gene set,

where *Y *represents the binary variable indicating the presence or absence of the treatment condition, *h *is a link function, for example the logit function, and intercept *α*. The *x*_*ij *_represents an element of the expression matrix of samples *i *and genes *j *and the regression coefficient *β*_*j *_for gene *j *(*j *= 1, 2, ..., *N*_*G*_). The Q-statistic for a gene set is given by

where R is the covariance matrix of the expression data and *μ*_2 _is the second central moment of *Y *under the null hypothesis. A high *Q *value is achieved when at least one *β*_*j *_is significantly different than zero. However, the Global Test makes a distributional assumption that the regression coefficients are from the same normal distribution which is unlikely to be true.

In this paper we develop a rank based test, the Rank Test that takes into account the magnitude of the intensity value as well as the correlation between genes within a specific pathway. The advantage of these tests is that no assumptions on distribution or independence are made. Genes in a pathway are first aligned by subtracting the median expression value for the combined treatment and control groups. The aligned expression values are then ranked within each subject and the vector of average ranks is calculated for each treatment. The distance between the two treatments is calculated and a permutation analysis is used to obtain a p-value for each pathway. The R Code for the Rank Test will be made available upon request. We also investigate a re-standardized version of the Rank Test (Modified Rank Test) where the observed distance is centered and scaled based on the mean and standard deviation from random subsets of genes of equal size.

Using two publicly available microarray datasets, we empirically evaluated Global and GSEA with the Rank Test and the Modified Rank Test. We also generated simulated data to test the reliability of each of the pathway analysis applications. Both real and simulated data were used to demonstrate that the rank based test has the highest, or nearly the highest power among the various techniques evaluated, especially when changes in the correlation structure of the pathway occurred. The rank based tests are robust and perform well under a wide range of assumptions.

## Results and Discussion

### Data Description

The Rank Test and the re-standardized Rank Test were compared with the Global test and with GSEA using two publicly available data sets. The first expression set is a mouse developmental toxicology experiment conducted by Dong et al. [14] using Agilent high-density oligonucleotide chips. The objective of the study was to investigate the effects of a thyroid disrupting chemical on the livers of developing pups. Pregnant dams were treated with 6-propyl-2-thiouracil (PTU) to produce hypothyroid pups. Livers were collected from control and PTU treated pups and RNA was labelled and hybridized to Agilent 22K arrays against a universal mouse reference RNA. The expression data for this experiment are available from the European Bioinformatics Institute (EBI) repository (accession number E-MEXP-1091). In the second dataset, Halappanavar et al. [15] investigated the effects of mainstream tobacco smoke (MTS) on global transcription in the mouse lung. Male C57B1/CBA mice were exposed to MTS from two cigarettes per day, 5 days/week, for 6 or 12 weeks. Agilent high density DNA microarrays were used to characterize global gene expression changes in whole lung. The data were retrieved from the National Centre for Biotechnology Information (NCBI) database (accession number GSE12930). We used Agilent arrays in the present experiment because this is the technology that we use in our facility. However, the findings of this work, and the algorithms developed, should apply to data from other DNA microarray platforms using different probe technologies.

### Data from EBI: E-MEXP-1091

In the Dong et al. [14] experiment, pregnant dams were rendered hypothyroid by treatment with 0.1% PTU in drinking water, from day 13 post-conception until weaning. Livers were collected from control and PTU treated pups at post-natal day (PND) 15. Each treatment group contained 5 males and 5 females. Treating gender as a block factor, we obtain two data sets, one for males and one for females. Analysis of the Agilent array consisted of 20651 probes which yielded 194 KEGG (Kyoto Encyclopedia of Genes and Genomes) pathways [16,17]. Pathways were constructed using the mgug4121a.db R library and consisted of only those pathways containing two or more genes with no missing expression values.

Using a MAANOVA [18] with an FDR corrected p-value of 0.10, Dong et al. [14] discovered 96 differentially regulated genes. Of these, 72 genes encoded proteins of known function. Approximately 50% (34 genes) belonged to various metabolism pathways (as expected as liver is the primary site of metabolism). A second large group of genes were part of development pathways (10 genes), as expected as the treatment was delivered across a broad developmental time frame. Using ONTO express [19], Dong et al. [14] found the most affected biological processes included metabolism, cell growth and maintenance, development, immune response, transcription, and signal transduction. However, no specific KEGG pathways were presented in the paper as affected by the treatment.

**Figure 1:**
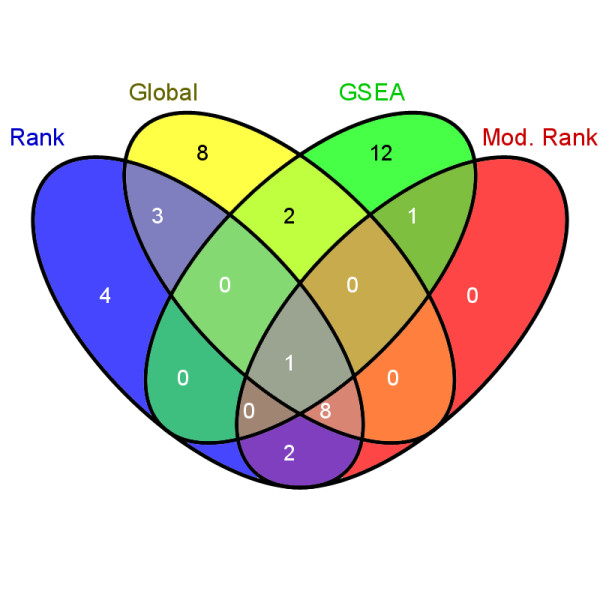
**Summary of the results from the male data for the 4 methods**.

**Figure 2:**
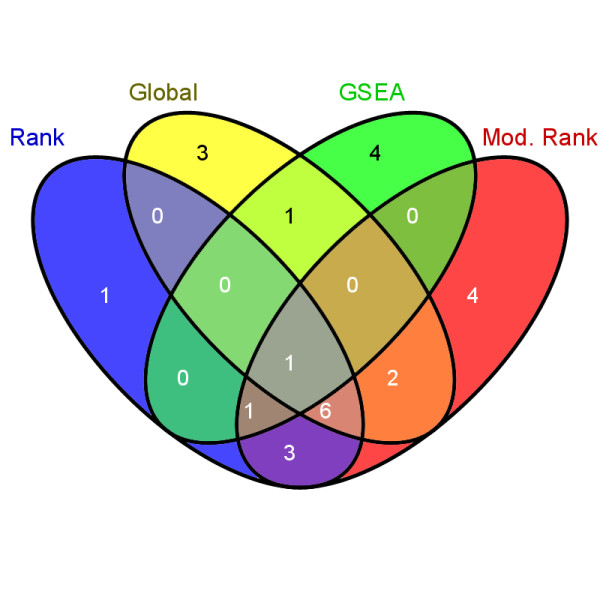
**Summary of the results from the female data for the 4 methods**.

We used the Dong et al. data to investigate the KEGG pathways that may be affected by the treatment. For the male and female expression sets, Global, GSEA, Rank and Modified Rank tests were applied to identify differences between the control and exposed groups for the 194 KEGG pathways. VENN diagrams using VENNY [20] were generated to compare the results within gender. The 0.01 significance level was chosen so that the Pre-Family Error Rate (PFE), i.e. the expected number of errors in the family, would be 1.94 for each method. Within males, a total of 41 pathways were significant, and 24 were unique to one of the 4 methods with only one common pathway (C21-Steroid hormone metabolism, Additional file 1: Table S1). Females exhibited 26 differential pathways, 12 of which were significant at the 0.01 level (Additional file 1: Table S1). The results for the male and female data are summarized in Figures 1 and 2.

Assuming no gender differences with respect to the identified pathways, VENN diagrams were generated. The striking observation is that GSEA had no common differential pathways for males and females, where the Global, Rank and Modified Rank methods had 7, 9, and 6 common pathways respectively. Of the 9 common pathways for the Rank test five contained at least one gene that was validated by RT-PCR in the Dong et al. study. For the Global method two of the seven pathways contained genes that were validated. For the Modified Rank test four of the six pathways contained at least one gene that was validated. Thus, a fair proportion of these pathways contain at least one gene validated by an alternative technology, providing some degree of confidence in the involvement of these pathways in response to PTU exposure.

The Endocrine signalling pathway, labelled GnRH was in Top 5 pathways ranked by p-value for GSEA in the male results. The liver is not the site of GnRH production and thus we don't expect that this pathway is affect per se. However, many of the genes that are found in this pathway are involved in numerous other signal transduction pathways. For example, the gene Egfr (epidermal growth factor receptor) is part of 12 other KEGG pathways, Protein Kinase C (Prkca) is a node in 23 other KEGG pathways, Ras is part of 20 (not including pathways related to human disease), Jun is part of 9, and various genes in the GnRH pathway that were differentially expressed are part of MAPK signalling pathways. This, in combination with the lack of change in the GnRH receptor itself, suggest that this pathway was found because of a broad level of change in signal transduction pathways, rather than a direct effect on GnRH signalling. Thus, the identification of this pathway is a good example demonstrating that responsive pathways need to be carefully scrutinized and biologically validated.

**Figure 3:**
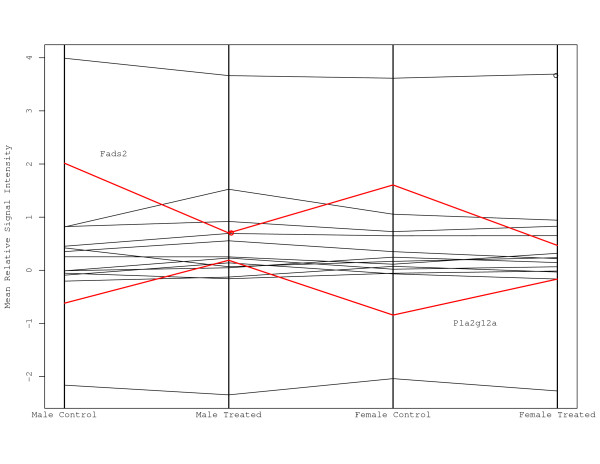
**C21-Steroid hormone metabolism pathway**. Parallel co-ordinate plots of the genes in the C21-Steroid hormone metabolism pathway are displayed. The genes Akr1c18 and Hsd3b1 are identified by red lines.

We generally observed two types of differences between control and treatment groups that affected gene rankings within pathways. Most gene set methods detected changes in mean intensities. For the C21-Steroid hormone metabolism pathway (Figure 3) of the 13 genes in the pathway, Akr1c18 and Hsd3b1 have fold changes less than -2.5 for both genders and for the males, Hsd11b1 and Hsd3b2 have fold changes greater than 1.6. This pattern implies that rankings of genes in these pathways diverge between control and treatment groups. However, the changes in this pathway in the female dataset were not of sufficient magnitude to be found significant by the GSEA test. Previous work has shown a significant increase in progesterone in the plasma of female rats that were rendered hypothyroid by chemical treatment (e.g., Tohei [21]). The primary gene involved in the maintenance of progesterone homeostasis is Akr1c18 (also known as 20-alpha-hydroxysteroid dehydrogenase). Dong et al.'s data indicate a 6-7 fold down-regulation in both males and females for this gene in hypothyroid livers of developing mice (Figure 1). Akr1c18 is specific to the C21 steroid hormone metabolism pathway. The other gene that shows consistent down-regulation in both sexes is 3-beta-hydroxy-delta 5-steroid dehydrogenase (Hsd3b1/Hsd3b2), which also participates in the maintenance of progesterone homeostasis. Additional genes in the C21 steroid hormone metabolism pathway are altered by the treatment, but show smaller fold changes or exhibit differences between males and females. However, two strong hits in this pathway in combination with published evidence in the literature demonstrating disruptions in progesterone levels as a result of altered thyroid hormone levels provide evidence to suggest that genes operating in this pathway are affected by the treatment either directly or indirectly in the livers of developing mice. Thus, we argue that the GSEA may have failed to identify C21 steroid hormone metabolism as an affected pathway.

**Figure 4:**
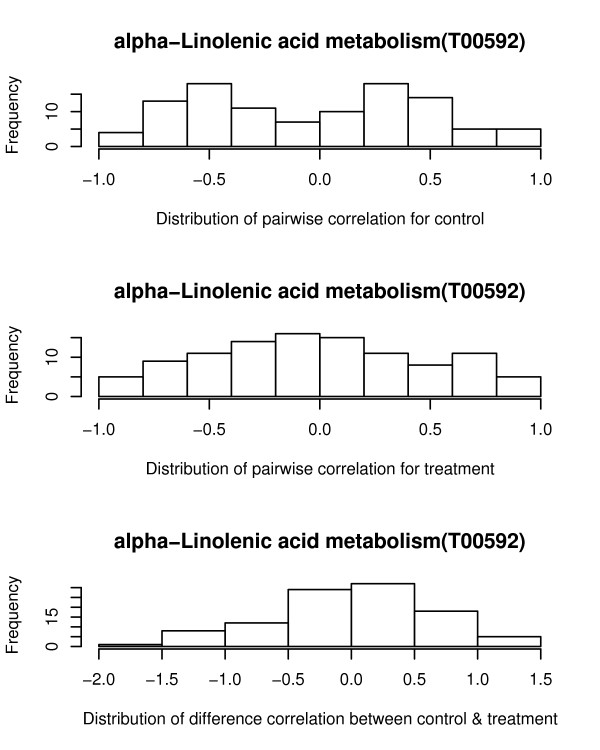
**Changes in gene correlations in the Alpha-Linolenic acid metabolism pathway**. Histograms of the gene correlations for the control and treated samples are presented with the histogram of the differences in correlation between the controls and treated.

A second type of difference between groups is due to change in the correlation between genes within a pathway. To examine the correlation between genes, the ranks of relative expression for each gene is established. Any two genes can be positively or negatively correlated or not correlated. One would expect that genes within a pathway to be correlated. If there is a shift in these correlations as a result of treatment this may affect this relationship among the genes. One example is the alpha-Linolenic acid metabolism pathway (Figure 4). The distribution of gene correlations for the control samples has two modes approximately at -0.5 and at 0.4. The distribution of gene correlations for the treated samples is different having a single mode close to 0. This suggests that there are more genes that are now correlated compared to the control distribution. The Rank Test identified a significant difference for the male and female datasets and the Global Test identified a significant difference for the males, but the other statistics failed to detect a significant difference. In both genders, Fads2 was greater than 2 fold down-regulated and Pla2g12a was greater than 1.5 up-regulated. Also, Acox1 for males was greater than 1.5 up-regulated. In terms of changes in correlations, Pla2g2c and Pla2g12b for the males samples had a spearman correlation of -0.8 for the controls and 0.6 for the treated samples, whereas for the females genes Pla2g4a and Pla2g2e had correlations of -0.9 and 0.8 for treated and controls. In all, there were 13 of 91 comparisons that had differences in correlation greater than 1.4. The distribution of correlations for the females also exhibited a similar pattern as in Figure 4 providing additional validation for disruption of this pathway. Disruptions in TH levels are known to result in alterations in fatty acid metabolism. A relationship between TH and alpha linolenic acid in the liver has previously been established [22,23]. Thus, the identification of this pathway is likely to be biologically-relevant.

The aim of the test that we have developed is to generate hypotheses based on microarray experimental data to assist in prioritizing follow-up experiments. Without extensive biological validation it is not possible to comment on whether these pathways have been correctly identified. Thus, we argue that the above pathways may be promising candidates for additional research. We describe one further biological investigation below, but a more powerful validation exercise is the simulation analysis described in the last section of this paper.

### Data from NCBI: GSE12930

Halappanavar et al. [15] used DNA microarrays to examine global transcriptional changes in whole lung tissues derived from mice exposed to mainstream tobacco smoke (MTS) for 6 or 12 weeks. From the MAANOVA analysis, 79 genes were either up- or down-regulated following MTS exposure. Among the 79 statistically differentially expressed genes cytochrome P450, family 1 (Cyp1a1), heme oxygenase (decycling)1 (Hmox1) and NAD(P)H dehydrogenase, quinine 1 (Nqo1) were validated using RT-PCR. In addition, cytokine interleukin 6 (IL-6) mRNA was upregulated alongside its antagonist, suppressor of cytokine signalling (SOCS3). These genes had also been reported in previous smoke-exposure studies. The authors examined IL-6 protein levels to confirm the finding, as well as some of its downstream targets. In this study, Pathway Studio (version-5, Ariadne Genomics Inc.) was used to identify pathways. From their analysis they identified a network of two core modules relating to the xenobiotic response pathway, and inflammation, cell survival and proliferation pathway.

The pathways identified at the 0.01 significance level by either the Rank Test, Global Test, GSEA and the Modified Rank test for the six week exposure were also identified by the respective methods in the twelve week exposure (Additional file 2: Table S2), with the exception of four pathways (Global: Basal transcription factors; Modified Rank Test: Glutamate metabolism, PPAR signalling pathway and Glioma). The Global test was the most sensitive statistic identifying 7 and 22 significant pathways followed by the Rank Test with 1, 17 and the Modified Rank Test with 6 and 11 significant pathways for the six and twelve week exposure respectively. The GSEA method did not identify any significant pathways. Of the 29 pathways declared significant, five pathways (Metabolism of xenobiotics by cytochrome P450, Tryptophan metabolism, Porphyrin and chlorophyll metabolism, Biosynthesis of steroids and Arachidonic acid metabolism) contained at least 1 gene that was RT-PCR validated in the Halappanavar et al. [15]. The gene Ptgs2 in the Arachidonic acid metabolism pathway was the only pathway that was unique to one method, the Global Test.

Thus, the general findings of the reanalysis of these publicly-available experimental data provide compelling support to the advantages of rank and global methods. However, without extensive RT-PCR validation, it is not possible to know if these methods identify more pathways, or are more accurate than the GSEA approach. As such, in the following sections we apply a simulation experiment to test these statistics.

### Simulation

The issues with empirical evaluations of methods are that we often do not know the underlying distributions of the data, i.e., the truth. Simulation is a tool often used to test new methodologies. Simulating data from known distributions allows us to measure how one method performs compared to another method under different scenarios.

The simulation conducted in this paper provides further evidence to support the ranking approach as a more sensitive method. In the proceeding sections we demonstrated that when the treatment and control samples come from the same population, the power of the rank test is higher. As well, we will evaluate the methods with respect to their ability to detect differences in mean, correlation or both.

For this simulation study only one pathway was generated. The number of probes in the pathway was set to 46 genes which is the average pathway size based on the E-MEXP-1091 dataset. Control samples were generated from a standard multivariate normal distribution N(0, I). We considered a variety of changes to characterize the treatment groups:

1) Mean change: the data were generated from the same distribution as the control samples except that the mean of the first three genes of the pathway was set to equal 2;

2) Correlation change: the data were simulated from a multivariate normal distribution N(0, Σ). The covariances between gene pairs in a pathway were set equal to 0.9 and the variances equal to 1;

3) Correlation and mean change.

For each of the above conditions, sample sizes of 5, 20, and 30 per group were used. The simulation was conducted as follows:

1) Data for the control and treated conditions for a given scenario and sample size were generated and then centered using the median.

2) The GSEA, Global and Rank Test tests were applied and p-values were obtained using 1000 random permutations (note that here the Modified Rank Test is equivalent to the Rank Test).

3) Significance was determined if the p-value from the test was less than or equal to 0.1.

For each scenario and sample size the above procedure was repeated 1000 times and the estimated power for each test was recorded in Table 1. A sample size of five was chosen as this is a typical sample size for most microarray experiments, while 20 and 30 replicates provide large sample properties of the statistic.

**Table 1:** Power calculations from the simulation study

		Sample Sizes per Group		
**Simulated Difference**	**Method**	**5**	**20**	**30**

No Change	Rank	0.117	0.102	0.095
	Global	0.107	0.051	0.056
	GSEA	0.092	0.120	0.112

Mean Change	Rank	0.739	1.000	1.000
	Global	0.853	1.000	1.000
	GSEA	0.230	0.777	0.950

Correlation Change	Rank	0.972	0.997	1.000
	Global	0.131	0.102	0.107
	GSEA	0.119	0.095	0.109

Mean and Correlation Change	Rank	1.000	1.000	1.000
	Global	0.422	0.993	1.000
	GSEA	0.075	0.053	0.050

Presented is the estimated power of the test for each method at varying sample sizes.

All three methods provide an error rate of approximately 0.1 in the no change case. The mean change condition the Global Test had the largest observed power (0.853) for a sample size of 5, followed by the Rank Test with a power of 0.739. The Rank and Global Test converged to a power of 1 at the 20 and 30 sample size. The GSEA method had the lowest power for this test case. Under the correlation change case, the Rank test out-performed the Global and GSEA methods. The Rank test achieved a power of 0.972 for the small sample size case whereas the other two methods had power estimates that resemble the no change test case for the small and large sample scenarios. When changes in the mean and correlation occurred, the power for the Rank Test was 1 for all sample sizes. The Global test outperformed GSEA and the power of the test approached 1 with increasing sample size. GSEA did not perform well when changes in correlation occurred, obtaining power estimates one would expect by chance. Considering the simulated conditions and sample sizes used the Rank Test was the most powerful test.

## Conclusions

Dimension reduction is critical in order to decipher underlying biological phenomena in microarray studies. Gene sets based on pathways or GO ontology provide an ideal approach to reduce the dimensionality through biological meaning. Working with pathways and gene sets that cover the probe sets on the microarray platform, rather than the individual probes, can dramatically reduce dimensionality and aid in biological interpretation. Using this approach, the investigator can evaluate datasets more readily compared to interpreting potentially long lists of differentially expressed genes.

We describe a non-parametric methodology to test whether or not a pathway exhibits a significant change compared to a control. The method revealed a large number of significantly changed pathways that were identified more efficiently and potentially more accurately than can be achieved by manually mining gene lists derived from standard analyses. Results for interesting pathways from this method are not impacted by their environments or surroundings, which happen in other existing methods (e.g. Fisher Exact's Test and GSEA). Moreover, our method takes into account changes in both the mean and in the correlation. The Rank and Modified Rank test demonstrate good performance on real as well as on simulated microarray data sets. However, the low degree of overlap between the approaches suggests that the use of more than one technique may be beneficial when conducting analyses of gene sets to avoid missing a novel result. In addition, the investigator is urged to continue to use alternative technologies (e.g., RT-PCR, protein analysis, etc.) to validate findings.

## Methods

### Normalization

For the empirical evaluation using the E-MEXP-1091 and GSE12930 datasets, the lowess approach [24] was used to normalize the data. Per-gene normalization was then performed centering the expression data by the median. Analysis was carried out on all genes regardless of flags.

### GSEA and Global

All analysis was conducted in R [25]. The Bioconductor [26] library and the GSEA 1.0 R package [5] were used. For the Global methodology, the Global test function in the Global test library was used to identify significant pathways.

### Rank Test

Assume that there are M genes belonging to a pathway. Subtract from each gene expression value, the median expression value obtained from the combined treatment and control groups. This process aligns the data thereby inducing subsequent analyses to be sensitive to changes in the mean. Next, for the *j*^*th *^subject in group *i*, let ω_*ij*_, represent the vector of ranks of the aligned intensity values of the *M *genes in the pathway. Set

The use of ranks serves two purposes. First, it captures for each subject, the correlation pattern of the aligned expression values. Second, it allows for a subsequent nonparametric analysis.

Motivated by the methods of Feigin and Alvo [27], we propose the test statistic

where prime indicates the transpose of the vector. Under the hypothesis that there is no change between the two groups, the statistic *S *should be small in magnitude. Let *S*_*obs *_be the value of the observed statistic.

Next, we propose a permutation test based on *S*. Under the null hypothesis that no change has occurred, the subjects in the two groups are interchangeable. Hence, we compute for each selection of *n*_*1 *_subjects from *n *a value of the statistic *S*. The nominal p-value is then given as

When the total number of possible permutations  is large, we randomly choose 1000 permutations among them.

### Modified Rank Test

The Rank test is defined independently of the other genes contained in the microarray. Efron and Tibshirani [28] considered two different hypotheses in connection with the problem of assessing the statistical significance of a pathway. The random null hypothesis states that the *M *genes in the pathway of interest have been chosen at random from the array. Hence, the null distribution of the test statistic is obtained by considering its value over all the possible sets of *M *genes in the array. On the other hand, to each subject corresponds an *M*-vector of expression values. The permutation hypothesis in that case states that the vectors are independent and identically distributed and hence, the distribution of the test statistic is obtained by permuting the vectors. As Efron and Tibshirani [28] point out, both hypotheses have shortcomings. The first tends to ignore correlations among the genes whereas the second does not take into account the array from which the genes are drawn. Instead, they proposed an adjusted statistic which re-standardizes the observed statistic *S*_*obs *_with mean m* and standard deviation σ* as follows:

where m*, σ* are the mean and standard deviation obtained by randomly selecting gene sets from the entire microarray and *m*_*s *_and σ_s _are the mean and standard deviation obtained by permutation of the labels for the specific pathway.

## Authors' contributions

The methodology for the proposed Rank Statistic was developed by MA. All analysis and code development was conducted by ZL. The biological interpretation of the case studies evaluated was conducted by CY. The manuscript was drafted and prepared by MA, ZL, AW and CY. MA was the overall team leader. All authors have read and approved the final manuscript.

## Supplementary Material

Additional file 1 **Table **1 **Significant Pathways identified in the E-MEXP-1091 Data sets**. A pathway is considered significant if the p-value of the test statistic is less than or equal to 0.01. Click here for file

Additional file 2 **Table 2 Significant Pathways discovered in GSE12930 Data Sets**. A pathway is considered significant if the p-value of the test statistic is less than or equal to 0.01. Click here for file
